# Estimating minimum dietary diversity for children aged 6–23 months: a comparison of agreement and cost of two recall methods in Cambodia and Zambia

**DOI:** 10.1017/S1368980024000107

**Published:** 2024-01-22

**Authors:** Laura S Hackl, Lidan Du-Skabrin, Amry Ok, Chiza Kumwenda, Navy Sin, Lukonde Mwelwa-Zgambo, Ramji Dhakal, Bubala Thandie Hamaimbo, Elise C Reynolds, Katherine P Adams, Charles D Arnold, Christine P Stewart, Erin M Milner, Sarah Pedersen, Jennifer Yourkavitch

**Affiliations:** 1 USAID Advancing Nutrition, Arlington, Virginia, USA; 2 John Snow Research and Training, Inc., 2733 Crystal Drive, 4^th^ floor, Arlington, Virginia 22202, USA; 3 SBK Research and Development, Phnom Penh, Cambodia; 4 School of Agricultural Sciences, Department of Food Science and Nutrition University of Zambia, Lusaka, Zambia; 5 Institute for Global Nutrition, University of California, Davis, CA, USA; 6 USAID Bureau for Global Health, Washington, DC, USA; 7 USAID Bureau for Resilience and Food Security, Washington, DC, USA; 8 Results for Development, Washington, DC, USA

**Keywords:** minimum dietary diversity, children, recall, method, Cambodia, Zambia

## Abstract

**Objective:**

To compare the agreement and cost of two recall methods for estimating children’s minimum dietary diversity (MDD).

**Design:**

We assessed child’s dietary intake on two consecutive days: an observation on day one, followed by two recall methods (list-based recall and multiple-pass recall) administered in random order by different enumerators at two different times on day two. We compared the estimated MDD prevalence using survey-weighted linear probability models following a two one-sided test equivalence testing approach. We also estimated the cost-effectiveness of the two methods.

**Setting:**

Cambodia (Kampong Thom, Siem Reap, Battambang, and Pursat provinces) and Zambia (Chipata, Katete, Lundazi, Nyimba, and Petauke districts).

**Participants:**

Children aged 6–23 months: 636 in Cambodia and 608 in Zambia.

**Results:**

MDD estimations from both recall methods were equivalent to the observation in Cambodia but not in Zambia. Both methods were equivalent to the observation in capturing most food groups. Both methods were highly sensitive although the multiple-pass method accurately classified a higher proportion of children meeting MDD than the list-based method in both countries. Both methods were highly specific in Cambodia but moderately so in Zambia. Cost-effectiveness was better for the list-based recall method in both countries.

**Conclusion:**

The two recall methods estimated MDD and most other infant and young child feeding indicators equivalently in Cambodia but not in Zambia, compared to the observation. The list-based method produced slightly more accurate estimates of MDD at the population level, took less time to administer and was less costly to implement.

Poor-quality diets are among the greatest obstacles to the survival, growth and development of children today^(1)^. Due to their rapid growth and development and small gastric capacity, infants and young children 6–23 months of age have very high nutrient requirements per unit body weight^(2)^. However, most children aged 6–23 months are not fed in alignment with global guidelines^(1)^.

Minimum dietary diversity (MDD) for children is a simple population-level indicator, commonly used to describe diet quality among children 6–23 month of age. It is defined as the proportion of children 6–23 months of age who receive foods from at least five out of eight defined food groups,^(3)^ and it is positively associated with dietary micronutrient adequacy and linear growth in young children^(4–6)^.

MDD has been routinely collected in numerous large-scale surveys using two types of 24-hour recall methods. The Demographic and Health Surveys Program uses a list-based recall method in which a list of food items is read to the respondent who indicates what the child consumed the previous day and night^(7)^. UNICEF’s Multiple Indicator Cluster Surveys and the US Government’s Feed the Future Zone of Influence (ZOI) Survey use a multiple-pass recall method comprising an open recall in which consumption of individual food items is recorded, followed by a list of items in food groups not mentioned in the open recall, followed by ‘Other solid, semi-solid or soft food?’ probes^(8)^.

Both methods capture information on food groups consumed on the previous day but do not take into account the portion size^(3)^. Recently, list-based and multiple-pass methods have been compared to weighed food records to assess MDD in women of reproductive age (MDD-W) in Cambodia, Ethiopia, and Zambia. The findings indicated both methods over-estimated the prevalence of MDD-W when compared to weighed food records^(9)^.

However, it is not known to what extent MDD estimates for children, when constructed from these two recall methods, agree with each other or with the observation. To address this evidence gap, USAID Advancing Nutrition designed a study comparing MDD indicator estimations derived from the two dietary recall methods with observed food intake. In addition, we also assessed measurement agreement for estimates of minimum acceptable diet defined as consuming at least the MDD and minimum meal frequency during the previous day AND are either breastfed or consumed the minimum milk feeding frequency during the previous day^(3)^. A secondary objective of the study was to assess the cost-effectiveness of each recall method, defined for this study as a comparison of the total cost of each method to the agreement of each method with the observation.

This study provides information for survey administrators, programme managers and data users about the relative practical merits and challenges with these two commonly used methods for dietary data collection.

## Methodology

### Study population and sampling

The sampling frame included all children aged 6–23 months in the Kampong Thom, Siem Reap, Battambang and Pursat provinces in Cambodia (mostly rural with a few peri-urban communities) and the Chipata, Katete, Lundazi, Nyimba and Petauke districts in Zambia (rural), which comprise the Feed the Future Phase I ZOI in each country^(10)^.

A target sample size of 578 was determined in each site aiming to detect a difference of 10 percentage points between two different recall methods – assuming a reference MDD prevalence of 30 per cent, alpha of 0·05, 80 % power, correlation of measures within subjects of 0·2 and a cluster design effect of two. Accounting for a 10 % attrition rate in Cambodia and 5 % in Zambia, we enrolled 636 and 608 households, respectively. Subsequently, we determined that an equivalence testing approach^(11)^ would be a more appropriate analysis. Specifically, the target sample size of 578 per study country would provide >80 % power to conclude that the two methods are equivalent with an equivalence limit of ± 10 percentage points.

In both countries, we employed two-stage sampling. First, we selected thirty enumeration areas within each ZOI using probability proportional to size (Stage 1), and then, we randomly selected twenty or twenty-one households per enumeration area based on a complete listing of all eligible households (Stage 2), (i.e. households with at least one child 6–23 months of age). In cases with more than one eligible participant in the household, only one was randomly selected. We excluded data for those participants for whom the study team did not complete the observation and both recall methods (see online supplementary material, Supplemental File 1).

In this study, the ‘participant’ was the eligible child. The ‘respondent’ was an adult of legal consenting age (18 years of age) or older who fed the participant on Day 1 of data collection (observation) and provided consent to participate in the study. The respondent would also be available for data collection via both recall methods on Day 2.

### Preparation for data collection

Prior to data collection, the multiple-pass recall instrument and food, recipe and ingredient lists were adapted to local contexts by the study personnel in each country, following standard guidance^(3)^. The list-based instrument was the infant and young child adaptation of the Diet Quality Questionnaire, which was already adapted for each country^(12)^ and made available to the study team in May 2022. All questionnaires were developed in English, translated into local languages (in Cambodia: Khmer; in Zambia: Chewa and Tumbuka), back-translated into English and revised for clarity by individuals not involved in the translation.

The research team programmed the observation form and questionnaires onto electronic tablets to allow the collection of data using the computer-assisted personal interview software. The devices used in Cambodia and Zambia were Kobo Toolbox Version 4.4, Cambodia; CSPro Version 7.7.3, USA, respectively. The software was programmed to perform data quality checks and capture interview durations.

Prior to the start of the study, we trained enumerators and supervisors in the survey methodology and data collection tools. Then, an enumerator visited the sampled households to inform the prospective study respondents and seek informed consent to participate in the study. After obtaining consent, the enumerator scheduled the in-home observations of food intake (Day 1) as well as times for the two recall methods on the subsequent day (Day 2). If the selected household was not available on the scheduled observation date, enumerators would make up to three attempts to visit this household until a replacement would be randomly selected.

### Data collection

Details of the list-based and the multiple-pass recall methods are described elsewhere^(3,12)^. Our study followed the standard application of these methods. The multiple-pass recall involves standard probing questions to help the respondent recall all foods and beverages that the child consumed the previous day and all reported foods consumed are recorded by enumerators. For mixed dishes, trained enumerators probe for the main ingredients of the dish, typically the primary two or three ingredients, as instructed in the WHO/UNICEF guideline^(3)^. The country-adapted Diet Quality Questionnaire lists of foods are publicly available for Cambodia and Zambia and designed for use without further adaptation (see online supplementary material, Supplemental File 2).

For each participant, three different enumerators collected children’s dietary data using the three different methods. The enumerators had their own tablets and were not aware of the results of the other data collection methods when collecting or uploading their data. On Day 1, one enumerator confirmed permission to spend the whole day observing from early in the morning (ideally, before the participant received their first food/meal) to evening (until the participant consumed the last food/meal). The enumerator inquired about foods consumed during the night and before s/he arrived at the household and also recorded all foods consumed by the participant during the observation period, as well as who prepared and administered the food.

On Day 2, the second and third enumerators interviewed the same respondent using the multiple-pass recall and the list-based recall in a random order with one administered in the morning and the other in the afternoon. If the respondent was not with the participant during part of Day 1, enumerators attempted to interview those individuals who fed the child during the periods in which the respondent was absent. If it was not possible to obtain this information, enumerators noted the absence of information. The enumerators inquired about foods consumed the night before last and all day yesterday until the child went to sleep, covering the same time period as the observation.

In Cambodia, we conducted data collection in June and July 2022, which is the rainy/monsoon season. In Zambia, data collection was conducted in March and April 2023, which is also the rainy season. The study included data collection across all days of the week, with weekdays and weekends proportionately represented to capture dietary patterns across the week.

### Statistical analysis

We analysed datasets from Cambodia and Zambia separately and accounted for the two-stage sampling design in all analyses. We estimated the prevalence of consumption for each of the eight food groups, MDD, minimum acceptable diet, minimum meal frequency and the mean dietary diversity score for both methods. Estimates from the two recall methods were compared for equivalence to the prevalence and scored based on the observation, using linear probability models while controlling for sequence of method administration following a two one-sided test equivalence testing approach.

Our primary objective was to determine if the two tools were equivalent to the reference in-home observation method in determining MDD. We defined equivalence as the two recall methods similarly classifying the majority of participants – such that the MDD prevalence estimates are within the pre-set equivalence margin of +/– 10 percentage points, when compared to the MDD prevalence estimated by the observation. As compared to common statistical methods for testing for differences, the null hypothesis in an equivalence testing approach is that the two groups are different. The 95 % CI of the difference must fall within the pre-specified equivalence margin. Consequently, a *P*-value <0·05 indicates a statistically significant finding of equivalence.

In addition, we calculated the sensitivity (correct classification of participants observed to achieve MDD), specificity (correct classification of participants observed to not achieve MDD) and per cent agreement of each method in comparison to the observation method. Agreement is the simple calculation of per cent of participants classified in the same way. We used Cohen’s kappa to measure agreement with scores of 0·21–0·40, 0·41–0·60, 0·61–0·80 and 0·81–1·00 as fair, moderate, substantial, and almost perfect agreement, respectively^(13,14)^. These measures of agreement quantify how well the assessment methods measure the individual’s actual intake on a given day, but they do not reflect the ability of the single-day’s recall to estimate usual intake over time.

For the scalar variable dietary diversity score, we also estimated intraclass correlation coefficients, interpreted as <0·5: poor, 0·5–0·75: moderate, 0·75–0·9: good, >0·9: excellent.

### Cost-effectiveness assessment

To evaluate the effectiveness of each method based on agreement, we defined a MDD prevalence agreement score specifically for this study. It is calculated for each method as 100 minus the percentage point deviation from the prevalence of MDD estimated by observation. For example, if the observation-based prevalence of MDD was 50 %, and the list-based prevalence of MDD was 60 %, the MDD prevalence agreement score for the list-based method would be 100–(60–50) = 90. Given that the MDD prevalence agreement score is a measure of how well each proxy method approximates the observation-based prevalence of MDD, it should be interpreted as a population-level measure of agreement. Then, we calculated the cost-effectiveness of each recall method by (1) estimating the method-specific total economic cost to prepare for, clean, collect and analyse the dietary intake data (including personnel costs, the opportunity cost of respondents’ time and non-personnel expenditures) and (2) dividing total economic costs by the MDD prevalence agreement score. Supplemental File 3 has details about cost inputs.

## Results

### Population characteristics

Table 1 shows the socio-demographics of the survey samples in both countries. In Cambodia, 52 % of the sampled children were male. The mean age of the participant children was 14·6 months. In Zambia, the number of male and female children sampled was almost the same (305 *v*. 303). The mean age was 14·4 months.

**Table 1:** Socio-demographic characteristics*

	Cambodia (*n* 636)		Zambia (*n* 608)	
	%	*n*	%	*n*
Child age, months				
Mean	14·6		14·4	
sd	5·2		5·0	
Child sex (% male)	52·0	331	49·8	303
Respondent’s relationship to the child				
Mother	81·1	516	93·1	566
Grandmother	16·2	103	5·8	35
Other	2·6	17	1·2	7
Respondent educational attainment				
Completed primary	44·2	281	62·1	338
Completed secondary	43·4	276	36·0	196
Respondent marital status (% married)	98·0	623	75·3	458
Respondent occupation				
Housewife	38·5	245	30·1	183
Farming	15·9	101	46·7	284
Day labour	11·8	75	3·6	22
Merchant	20·0	127	10·0	61
Other	13·8	88	9·6	58
Household characteristics				
Improved sanitation†	81·9	521	35·8	218
Improved drinking water†	100	636	87·8	534
Clean cookstove use‡	36·0	229	2·2	13
Improved roof materials§	98·3	625	76·2	463
Improved flooring§	44·3	282	53·2	323
Improved wall materials§	51·6	328	75·8	460

* Values represent mean ± sd or % (*n*)

† Improved sanitation and water quality defined using the WHO/UNICEF Joint Monitoring Programme definitions^(20)^. Improved sanitation facilities include flush/pour flush toilets connected to piped sewer systems, septic tanks or pit latrines; pit latrines with slabs (including ventilated pit latrines) and composting toilets. Improved drinking water sources include piped water, boreholes or tube wells, protected dug wells, protected springs, rainwater and packaged or delivered water.

‡ Clean cookstoves were defined using the WHO Clean Household Energy Solutions Toolkit 9^(21)^ criteria and include solar, electric, biogas, natural gas, liquefied petroleum gas and alcohol fuels including ethanol.

§ Improved roof materials include metal, cement, asbestos and iron/roofing sheets. Improved floor materials include ceramic tiles, cement and carpet. Improved wall materials include cement, stone with lime/cement, bricks, cement blocks and mudbrick.

In Cambodia, 81 % of respondents were the participants’ mothers, followed by grandmothers (16·1 %), and 98 % of the respondents were married. Close to 40 % reported being housewives while another 40 % were engaged in some form of work – merchant (20·0 %), farming (15·9 %) and day labour (11·8 %). Over 90 % (92·5 %) reported receiving at least primary education and 43·4 % receiving secondary education.

In Zambia, 93 % of respondents were the participants’ mothers and 75 % were married. About one-third (30 %) of respondents were housewives, and nearly half (46·7 %) worked in farming with smaller percentages working as merchants (10·0 %) or day labourers (3·6 %). Almost all respondents attended school, with 99·8 % receiving at least primary education and 36 % receiving secondary education.

Notably, a greater percentage of respondents in Cambodia reported using improved drinking water sources (100 % *v*. 87. 8 % compared to Zambia), improved sanitation sources (81·9 % *v*. 35·8 %) and clean cookstoves (36·0 % *v*. 2·2 %).

### Dietary diversity indicators

The percentage of children attaining MDD based on the in-home observation in Cambodia was 29·4 % and 58·2 % in Zambia (Table 2). In both countries, almost all children were fed at minimum meal frequency; therefore, the prevalence of minimum acceptable diet was similar to that of the MDD. Food groups more frequently observed in Cambodia than in Zambia included dairy products (54·2 % *v*. 5·2 %) and flesh foods (93·9 % *v*. 26·9 %). Food groups more frequently observed in Zambia than in Cambodia included breast milk (77·9 % *v*. 61·1 %), beans, peas, lentils, nuts and seeds (69·8 % *v*. 6·5 %), eggs (20·0 % *v*. 6·5 %), vitamin A-rich fruits and vegetables (69·2 % *v*. 32·0 %), and other fruits and vegetables (93·3 *v*. 46·1 %). Nearly universally, we observed grains, roots, tubers and plantains in both settings.

**Table 2:** Comparison of the proportions of children who were reported to have consumed each food group and who achieved minimum dietary diversity using observation and the two methods

	Cambodia					Zambia				
	Observation	List-based		Multiple pass		Observation	List-based		Multiple pass	
	%	%	Equivalence *P*-value*	%	Equivalence *P*-value*	%	%	Equivalence *P*-value*	%	Equivalence *P*-value*
Breast milk	54·2	61·1	0·002	66·7	0·92	77·9	78·4	<0·001	79·9	<0·001
Grains, white/pale starchy roots, tubers, and plantains	93·9	94·8	<0·001	96·1	<0·001	99·3	98·9	<0·001	99·4	<0·001
Beans, peas, lentils, nuts, and seeds	6·5	6·0	<0·001	7·7	<0·001	69·8	69·9	<0·001	72·2	<0·001
Dairy products (milk, infant formula, yogurt, cheese)	54·2	61·1	0·002	66·7	<0·001	5·2	6·4	<0·001	9·3	<0·001
Flesh foods (meat, fish, poultry, organ meats)	93·9	94·8	<0·001	96·1	<0·001	26·9	35·3	0·476	35·3	0·082
Eggs	6·5	6·0	<0·001	7·7	<0·001	20·0	25·7	0·176	25·9	0·047
Vitamin A-rich fruits and vegetables	32·0	32·3	<0·001	34·7	<0·001	69·2	79·5	0·512	84·1	0·990
Other fruits and vegetables	51·2	46·1	0·001	49·8	<0·001	93·3	88·9	<0·001	94·3	<0·001
Minimum dietary diversity	29·4	30·8	<0·001	36·7	0·04	58·2	62·2	0·095	68·4	0·537
Minimum meal frequency	99·4	100·0	<0·001	100·0	<0·001	99·9	91·7	0·168 †	94·4	0·024
Minimum acceptable diet	29·4	30·8	<0·001	36·7	0·04	58·2	60·0	0·037	66·5	0·197
Dietary diversity score			<0·001		<0·001			<0·001		<0·001
Mean	3·79	3·83		4·00		4·6	4·8		5·0	
sd	1·31	1·29		1·34		1·0	1·1		1·1	

* *P*-values less than 0.05 are considered significant. A significant finding in this case means the proxy method performed equally as well (within 10 percentage points) as the in-home observation in estimating population prevalence for the given indicator.

† Unable to control for sequence of tools due to only 1 observation not meeting minimum meal frequency in observation tool

In Cambodia, both the list-based and the multiple-pass recall produced estimates of MDD that were within the equivalence margins of the in-home observations, although the estimated prevalence using the multiple-pass method was marginally higher (36·7 %) compared to the in-home observation (29·4 %). The prevalence of reported breast milk consumption using the multiple-pass recall (66·7 %) was not equivalent to that of the in-home observation (54·2 %).

In Zambia, both the list-based and multiple-pass recalls produced over-estimates of MDD (62·2 % from the list-based recall and 68·4 % from the multiple-pass recall compared to 58·2 % from the in-home observation). Notably, both recall methods over-estimated the prevalence of consumption of flesh food, eggs and vitamin A-rich fruits and vegetables.

### Method performance characteristics

In both countries, the multiple-pass recall was more sensitive (>90 %) than the list-based recall to correctly classify participants observed to achieve MDD (93·7 % *v*. 78·6 % in Cambodia, 90·0 % *v*. 80·4 % in Zambia) (Table 3). By contrast, specificity was more moderate for both recall methods in Zambia (63·2 % for list-based *v*. 61·7 % for multiple-pass recall) but was high in Cambodia (89·1 % *v*. 87·1 %). In addition, there were low specificities and high sensitivities for a few food groups that were very frequently consumed – grains, roots, tubers and plantains in both countries and other fruits and vegetables in Zambia. The multiple-pass recall had a better combination of sensitivity and specificity for estimating consumption of beans, peas, lentils, nuts and seeds; flesh foods; and eggs in both countries.

**Table 3:** Comparison of performance characteristics of each recall method to the observation

	Cambodia (%)		Zambia (%)	
	List-based	Multiple pass	List-based	Multiple pass
Minimum dietary diversity				
Sensitivity	78·6	93·7	80·4	90·0
Specificity	89·1	87·1	63·2	61·7
Per cent agreement	86·0	89·0	73·2	78·2
Cohen’s Kappa (0–1)	0·67	0·75	0·44	0·54
Minimum meal frequency				
Sensitivity	100	100	91·7	94·4
Specificity	0	0	0	0
Per cent agreement	99·4	99·4	91·6	94·3
Cohen’s Kappa (0–1)	0·0	0·0	0·00	0·00
Minimum acceptable diet				
Sensitivity	78·6	93·7	78·0	88·0
Specificity	89·1	87·1	65·0	63·4
Per cent agreement	86·0	89·0	72·6	77·7
Cohen’s Kappa (0–1)	0·67	0·75	0·43	0·53
Dietary Diversity				
Interclass correlation coefficient	0·82	0·86	0·72	0·75
Cohen’s Kappa	0·54	0·61	0·29	0·37
Breast milk				
Sensitivity	100	99·7	99·2 %	99·3 %
Specificity	84·9	72·4	95·0	88·4 %
Per cent agreement	93·1	87·2	98·3	96·9 %
Cohen’s Kappa (0–1)	0·86	0·74	0·95	0·91
Grains, white/pale starchy roots, tubers, and plantains				
Sensitivity	98·3	99·7	99·2	99·8
Specificity	59·4	59·4	37·6	66·3
Per cent agreement	96·0	97·2	98·3	98·7
Cohen’s Kappa (0–1)	0·62	0·71	0·29	0·70
Beans, peas, lentils, nuts, and seeds				
Sensitivity	53·8	92·6	86·1	91·9
Specificity	97·3	98·1	67·7	73·1
Per cent agreement	94·5	97·8	80·5	86·2
Cohen’s Kappa (0–1)	0·53	0·83	0·54	0·67
Dairy products (milk, infant formula, yogurt, cheese)				
Sensitivity	96·4	98·4	67·8	81·0
Specificity	95·8	95·2	96·9	94·6
Per cent agreement	96·1	96·7	95·4	93·9
Cohen’s Kappa (0–1)	0·92	0·93	0·58	0·55
Flesh foods (meat, fish, poultry, organ meats)				
Sensitivity	93·3	98·4	82·5	87·7
Specificity	88·1	92·3	82·1	84·0
Per cent agreement	91·4	96·4	82·2	85·0
Cohen’s Kappa (0–1)	0·81	0·92	0·59	0·65
Eggs				
Sensitivity	92·6	96·9	85·6	89·1
Specificity	95·3	97·0	89·3	89·9
Per cent agreement	94·6	97·0	88·5	89·8
Cohen’s Kappa (0–1)	0·86	0·92	0·68	0·71
Vitamin A-rich fruits and vegetables				
Sensitivity	86·8	95·2	92·6	97·2
Specificity	93·3	93·7	50·0	45·3
Percent agreement	91·2	94·2	79·5	81·2
Cohen’s Kappa (0–1)	0·80	0·87	0·47	0·49
Other fruits and vegetables				
Sensitivity	81·2	91·4	91·3	97·4
Specificity	90·7	93·9	44·7	48·8
Per cent agreement	85·8	92·6	88·2	94·1
Cohen’s Kappa (0–1)	0·72	0·85	0·28	0·50

The multiple-pass recall method agreed more with the observation, i.e. it was able to accurately classify a higher proportion of participants as meeting MDD than the list-based recall in both Cambodia (89 *v*. 86 %) and Zambia (78 *v*. 73 %). Cohen’s Kappa, which measures agreement, was substantial in Cambodia (0·67 for list-based *v*. 0·75 for multiple pass), but only moderate in Zambia (0·44 *v*. 0·54). It was almost always higher for the multiple-pass recall compared to the list-based recall, which means that the multiple-pass method was able to correctly identify a higher proportion of the same individual participants who met MDD, also identified by the observation, while the list-based recall did not agree with the observation as closely. This difference also explains the variation in *p*-values (Table 2) where the prevalence of consumption estimated from both methods is similar.

### Cost-effectiveness

Table 4 presents the estimates of the total economic cost to prepare for, collect, clean and analyse the dietary data and the cost-effectiveness of each method. In Cambodia, the multiple-pass recall method cost more ($7 more per participant) than the list-based method ($82 *v*. $75), which was primarily attributable to higher personnel costs to prepare for data collection (computer-assisted personal interview development, translation of questionnaires, etc.) using the multiple-pass recall. In Zambia, the multiple-pass recall method also costs more ($5 more per participant) than the list-based method ($91 *v*. $86), driven primarily by higher personnel costs associated with data collection and supervision.

**Table 4:** Personnel and total costs, and cost-effectiveness by activity and recall method

	Cost to complete activity (2022 US dollars)					
	Cambodia (*n* 636)			Zambia (*n* 608)		
Activity	List-based	Multiple pass	Difference†	List-based	Multiple pass	Difference†
Preparation for data collection*	2955	4105	1150	3252	3347	95
Training	6843	6900	58	2190	2190	0
Piloting	5363	5678	315	2040	2027	−13
Data collection and supervision	15 498	15 505	8	15 504	17 811	2307
Data monitoring	2825	3105	280	1267	1267	0
Data cleaning	4700	5050	350	396	396	0
Data preparation and analysis	657	849	192	468	605	137
Personnel costs	38 840	41 192	2352	25 117	27 643	2526
Personnel costs per participant	61	65	4	41	45	4
Expenditures‡ and overhead	8689	10 690	2001	27 180	27 331	151
Value of respondents’ time	268	357	89	142	284	142
Total cost	47 796	52 239	4442	52 439	55 257	2819
Total cost per participant	75	82	7	86	91	5
Minimum dietary diversity prevalence agreement score §	99	93	−6	96	90	−6
Cost-effectiveness (2022 USD) ||	485	564	79	546	615	69

* Preparation for data collection: hiring personnel, computer-assisted personal interview development, questionnaire translation, etc.

† Difference calculated as cost for multiple-pass recall minus cost for the list-based method.

‡ Includes expenditures on venues, supplies, equipment, participant gifts, transportation, etc.

§ The research team calculated the minimum dietary diversity prevalence agreement score for each method as 100 minus the percentage point deviation from the prevalence of minimum dietary diversity estimated by observation.

|| Cost-effectiveness defined as cost per unit of agreement based on the minimum dietary diversity prevalence agreement score.

In both countries, because the prevalence of MDD estimated by list-based recall was closer to that estimated by the observation, the list-based method had a higher MDD agreement score. That, combined with the lower cost of the list-based method, resulted in the list-based method being more cost-effective than the multiple-pass method in both countries ($79 less per unit of agreement based on the MDD prevalence agreement score in Cambodia and $69 less per unit of agreement in Zambia).

In both countries, it took longer to administer the multiple-pass questionnaire than the list-based questionnaire per participant, approximately 8 min longer in Cambodia (22:10 and 13:55) and 11 min longer in Zambia (16:54 and 05:39) (see online supplementary material, Supplemental File 3).

## Discussion

In this study in Cambodia and Zambia, we found that the measurement agreement of MDD prevalence estimation varied by country and by recall method. In Cambodia, both recall methods were equivalent to the in-home observation, although the estimated prevalence using the multiple-pass recall method was marginally higher (36·7 %) compared to the in-home observation (29·4 %). The primary reason for this over-estimation by the multiple-pass method was due to more frequent reporting of breast milk consumption than was observed. However, in Zambia, both the list-based and multiple-pass recall methods significantly over-estimated the prevalence of MDD, due to the over-estimation of consuming foods from the flesh foods, eggs and vitamin A-rich fruits and vegetables food groups. The multiple-pass method was more sensitive, i.e. accurately classified the participants who met the MDD than the list-based recall in both countries, although both had high sensitivity. Both methods had high specificity in Cambodia, but only moderate specificity in Zambia. In both countries, it costs less to prepare for, collect and analyse the dietary data using the list-based method, and the list-based method yielded estimates of MDD prevalence closer to the MDD prevalence based on observations. Since minimum meal frequency was nearly 100 % in both countries, minimum acceptable diet was nearly identical to MDD.

This study provides important information about the performance and relative costs of two common recall methods to estimate MDD and other infant and young child feeding indicators. Our findings showed that foods recorded via the multiple-pass method agreed with the observation more often than those recorded via the list, but the multiple-pass method costs more to implement in both countries. The list-based recall method was more cost-effective than the multiple-pass method in both countries. These findings have implications for data collection efforts concerned with producing population-level estimates of nutrition indicators for children.

Although some studies of women and older children found reporting errors of omission of items consumed to be more frequent than over-reporting consumption^(15–17)^, in Zambia, we found consistent over-reporting of young children’s consumption of flesh foods, egg and vitamin A-rich fruits and vegetables from both methods. These foods are regularly promoted to improve children’s diets, and it is likely that social desirability bias affected our results (i.e. respondents reported what they knew to be correct rather than what they actually consumed)^(18)^. Our speculation is supported by the fact that the study area not only receives Feed the Future ZOI interventions, it is also part of the Government of Zambia’s Scaling Up Nutrition Programme (first 1000 Most Critical Days Programme phase II)^(19)^. In fact, a similar pattern of over-reporting was found in the MDD-W study data from Zambia^(9)^.

For nutrition programmes that aim to promote consumption of certain foods, we note that the list-based method does not provide as detailed information as the multiple-pass method, and those details about specific foods may be important to monitor. Regardless of the recall method used, we urge caution about the interpretation of dietary indicators in the context of social and behavioural change programmes promoting particular dietary patterns or consumption of particular food groups due to the likelihood of social desirability bias in responses to dietary recalls. In addition, neither recall method is recommended for estimating individual-level dietary patterns due to random within-person error and day-to-day variability in dietary intake. Random errors will reduce both sensitivity and specificity of the assessment instrument.

Hanley-Cook et al. compared list-based and multiple-pass recalls to a weighed food records to estimate MDD-W and found that women over-reported achieving MDD-W using both methods^(9)^. They concluded that the list-based method yielded MDD-W estimates further from the observation than the multiple-pass method (16 % and 10 %, respectively). That study used convenience sampling and a different type of analysis than our study, which might partly account for the different findings.

While we also found that both methods over-estimated MDD, the estimates yielded by both recalls were statistically equivalent in Cambodia, while in Zambia they were not. We observed a tendency to over-report consumption of all food groups, with the multiple-pass method reporting higher consumption than the list-based method, especially for dairy. The opportunity to recall multiple times, with prompting, may support the over-reporting of consumption.

This study has several strengths. To our knowledge, this is the first study to compare MDD derived from two recall methods to an observation. The children who participated in the study were representative of a defined region (ZOI) and so we were able to provide a representative, regional estimate to programme planners and policymakers. The sample sizes were large enough to detect statistically significant differences. We also assessed and compared relative costs of using recall methods to aid decisions regarding use. We minimised systematic error through supporting locally-led data collection with trained and tested enumerators and supervisors^(17)^. We followed standard guidelines for adapting the multiple-pass recall method^(3)^ and used the infant and young child Diet Quality Questionnaire without further adaptation, as instructed^(12)^. We minimised random errors through standard quality control processes^(17)^ including daily checks by supervisors and programming the computer-assisted personal interview to avoid implausible or certain erroneous values. We had low to no drop-outs (see online supplementary material, Supplemental File 1).

This study also has several limitations. The list-based questionnaires were updated during the research period; the version we used in Zambia included questions about breast-feeding after birth and the wording caused some confusion to respondents, which might have increased administration time. In addition, the inclusion of ifisashi (any mixed dish comprising of groundnut flour as one of the main ingredients, other ingredients include all sorts of dark green leafy vegetables, sweet potatoes, pumpkins, samp, okra, or small fish like kapenta) as a food item on the list was confusing for enumerators. These issues were corrected in the current version of the tool that is available online.

In estimating the total cost of each method, because the list-based survey instruments were already adapted to each country while the multiple-pass instruments required adaptation, we did not include adaptation costs in our total cost estimates. However, with the creation of the standardised Diet Quality Questionnaire food lists and subsequent elimination of the need for country-specific adaptation when using that list-based method, it could be argued that adaptation costs should not be included for the list-based recall, but should be included for the multiple-pass method, particularly when implementation of the multiple-pass method includes collection of individual foods. If we had included the time needed to adapt the multiple-pass questionnaire in our total cost estimate for implementing the multiple-pass method, the difference in cost between the two methods would have been even larger, and the relative cost-effectiveness of the list-based method would have been enhanced. It is also worth noting that the programming of the computer-assisted personal interview software was different in both countries which could have caused differences in determining the start and stop times (which would have more effect on the multiple-pass than on the list-based method). Moreover, some personnel did not track their time in the moment of data collection and team members had to estimate it retrospectively. Since we collected the dietary data in the context of a research project, our cost estimates may be higher than they would have been otherwise. Finally, it is important to note that these findings are based on an application of the multiple-pass method using an open recall, in which individual foods consumed were recorded in the data. This added to the cost (for programming tablets to record individual foods and for time spent collecting the data) but also likely impacted the degree of agreement between that method and the observation. The relative cost and agreement of the multiple-pass method may be different if data on individual foods were not collected.

Regarding our statistical analysis, we used a 10-percentage point equivalence margin to compare the recall methods and the observation. We acknowledge that a programme manager or policymaker may be concerned with smaller differences, especially for monitoring changes. Given the differences observed between recall methods, the same method should be consistently used for monitoring changes over time.

### Conclusion

The performance of the recall methods to estimate MDD prevalence varied by country and by method. Both methods were equivalent to the observation in Cambodia but neither was equivalent in Zambia. The list-based estimates of MDD prevalence were closer to the true population prevalence based on the observation. The list-based recall method was also more cost-effective than the multiple-pass method in estimating population-based indicators. This study provides important information about the performance and relative costs of two common recall methods to estimate MDD and other infant and young child feeding indicators.

## Supporting information

Hackl et al. supplementary material Hackl et al. supplementary material

## Supplementary material

For supplementary material accompanying this paper visit https://doi.org/10.1017/S1368980024000107
