# Supplementary material for: Estimating minimum dietary diversity for children aged 6–23 months: a comparison of agreement and cost of two recall methods in Cambodia and Zambia
Source: Public Health Nutr. 2024 Jan 22;27(1):e79. doi: 10.1017/S1368980024000107 (PMC10966926; doi:10.1017/S1368980024000107)
Supplement: Hackl et al. supplementary material [file S1368980024000107sup001.docx]

**Supplemental File 1. Study flow diagrams**

**Cambodia**

Observation (n =638)

Loss (n = 2)

Random allocation

Proxy method 1

List-based in the morning (n = 289)

Proxy method 2

Day 1

Day 2

Multiple-pass in the afternoon (n = 290)

Multiple-pass in the morning (n = 346)

List-based in the afternoon (n = 347)

**Zambia**

Observation (n = 608)

Loss (n = 0)

Random allocation

Proxy method 1

List-based in the morning (n = 285)

Proxy method 2

Day 1

Day 2

Multiple-pass in the afternoon (n = 292)

Multiple-pass in the morning (n = 316)

List-based in the afternoon (n = 323)

**Supplemental File 2. Forms and Questionnaires**

**Data Collection Form for Observation on Day 1**

**DAY 1: Confirmation of eligibility**

**Observer name:**

**Date:**

**Day of week:**

**Location:**

**Household ID:**

**TIME BEGAN: Timestamp**

1. Did respondent provide verbal consent to participate? Y/N
2. What is respondent’s relationship to child? Text
3. What name was given to the baby?
4. Is (NAME) a boy or a girl? Boy/Girl
5. On what day, month, and year was (NAME of child) born? DD/MM/YYYY
6. Is today a special day/holiday? Y/N

***NOTE: IF CHILD IS NOT 6–23 MONTHS OF AGE, OR IF TODAY IS A FEAST DAY OR PART OF AN EXTENDED FAST, THEN END INTERVIEW***

**Demographic information**

[Note to the interviewer: These questions were taken from the [DHS Phase-8 Survey](https://dhsprogram.com/publications/publication-DHSQ8-DHS-Questionnaires-and-Manuals.cfm) Household and Women’s modules (DHS 2020). Additional questions may include questions regarding domestic staff, house/land ownership, and number of individuals per sleeping room. Response options and exact wording for these questions may slightly differ between countries and will also be discussed with local partners].

| **Question** | **Response Options** |
| --- | --- |
| **Household Module**  **Respondent’s relationship to child:** Text | |
| What is the main source of drinking water for members of your household? | **Piped water**  Piped into dwelling  Piped to yard/plot  Piped to neighbor  Public tap/standpipe  Tube well or borehole  **Dug well**  Protected well  Unprotected well  **Water from spring**  Protected spring  Unprotected spring  Rainwater  Tanker truck  Cart with small tank  Surface water (river/ dam/ lake/ ponds/ stream/ canal/ irrigation channel)  Bottled water  Other |
| What kind of toilet facility do members of your household usually use? | **Flush or pour flush toilet**  Flush to piped sewer system  Flush to septic tank  Flush to pit latrine  Flush to somewhere else  Flush, don’t know where  **Pit latrine**  Ventilated improved pit latrine  Pit latrine with slab  Pit latrine without slab/open pit  Composting toilet  Bucket toilet  Hanging toilet/hanging latrine  No facility/bush/field  Other |
| In your household, what type of cookstove is mainly used for cooking? | Electric stove  Solar cooker  Liquefied petroleum gas (LPG)/cooking gas stove  Piped natural gas stove  Biogas stove  Liquid fuel stove  Manufactured solid fuel stove  Traditional solid fuel stove  Three stone stove/open fire  No food cooked in household  Other |
| At night, what does your household mainly use to light the home? | Electricity  Solar lantern  Rechargeable flashlight, torch or lantern  Battery powered flashlight, torch or lantern  Biogas lamp  Gasoline lamp  Kerosene or paraffin lamp  Charcoal  Wood  Straw/shrubs/grass  Agricultural crop  Animal dung/waste  Oil lamp  Candle  No lighting in household  Other |
| How many rooms in this household are used for sleeping? | Number of rooms: |
| Does your household have:  Electricity  A radio  Television  Non-mobile telephone  Computer  Refrigerator  Additional items  Additional items for Zambia include—  Access to internet  Bed  Table  Sofa  Washing machine  Air conditioner  Generator  Microwave  Geyser  Grain grinder  Plough  Tractor  Hammer mill  Additional items for Cambodia include—  Wardrobe  Sewing machine or iron  CD/ DVD player  generator/battery/solar panel | Yes/No (Y/N) for each item |
| Does your household own—  Watch  Mobile phone  Bicycle  Motorcycle or motor scooter  Animal-drawn cart  Car or tuck  Boat with motor  Additional items for Zambia include—  Banana boat  Additional items for Cambodia include—  Bicycle/cyclo  Motorcycle-cart  Tractor/Koyon (two-wheel local-made tractor) | Y/N |
| Does any member of this household have an account in a bank or other financial institution? | Y/N |
| Does any member of this household use a mobile phone to make financial transactions such as sending or receiving money, paying bills, purchasing goods or services, or receiving wages? | Y/N |
| Main material of the floor of the dwelling | **Natural floor**  Earth/sand (Additional option for Cambodia: Clay)  Dung  **Rudimentary floor**  Wood planks  Palm/bamboo  **Finished floor**  Parquet or polished wood  Vinyl or asphalt strips  Ceramic tiles  Cement  Carpet  Other (specify)  Additional option for Cambodia: Floating house |
| Main material of the roof of the dwelling | **Natural roofing**  No roof  Thatch/palm leaf (Cambodia: bamboo/ thatch/palm leaf)  Sod  **Rudimentary roofing**  Rustic mat  Palm/bamboo  Wood planks  Cardboard  **Finished roofing**  Metal  Wood  Calamine/cement fiber  Ceramic tiles  Cement  Roofing shingles  Other  Additional option for Zambia: Asbestos  Additional option for Cambodia: Plastic sheet |
| Main material of the exterior walls of the dwelling | **Natural walls**  No walls  Cane/palm/trunk  Dirt  **Rudimentary walls**  Bamboo with mud  Stone with mud  Uncovered adobe  Plywood  Cardboard  Reused wood  **Finished walls**  Cement  Stone with lime/cement  Bricks  Cement blocks  Covered adobe  Wood planks/shingles  Other  Additional options for Zambia: Mudbrick  Additional options for Cambodia:  Palm/bamboo/thatch  Metal  Covered adobe |
| How many of the following animals does this household own?  Traditional cattle  Dairy cattle  Horses/donkeys/mules  Goats  Sheep  Chickens  Pigs  Rabbits/other poultry  Others (specify) | Number of animals |
| In the last month, has there been any time when your household did not have sufficient quantities of drinking water when needed? | Y/N/Don’t know (DK) |
| **Women’s Module** | |
| Have you ever attended school? | Y/N |
| What is the highest level of school you attended: primary, secondary, or higher? | Primary  Secondary  Higher |
| What is the highest [GRADE/FORM/YEAR] you completed at that level? | Grade/Form/Year |
| What is your religion? | [Response options for Cambodia]  Buddhism  Muslim  Christianity  Other |
| What is your ethnic group? | [Response options for Cambodia]  Khmer  Cham  Others |
| Are you currently married or living together with a man as if married? | Yes, currently married  Yes, living with a man  No, not in union |
| What is your marital status now: are you widowed, divorced, or separated? | Widowed  Divorced  Separated |
| Is your (husband/partner) living with you now or is he staying elsewhere? | Living with her  Staying elsewhere |
| Does your (husband/partner) have other wives or does he live with other women as if married? | Y/N/DK |
| What is your main occupation? That is, what kind of work do you mainly do? | Text |
| Aside from your own housework, have you done any work outside your home in the last 7 days? | Y/N |
| As you know, some women take up jobs for which they are paid in cash or kind. Others sell things, have a small business or work on the family farm or in the family business. In the last 7 days, have you done any of these things or any other work? | Y/N |
| Although you did not work in the last 7 days, do you have any job or business from which you were absent for leave, illness, vacation, maternity leave, or any other such reason? | Y/N |
| Have you done any work outside your home in the last 12 months? | Y/N |
| Do you do this work for a member of your family, for someone else, or are you self-employed? | For family member  For someone else  Self-employed |
| Do you usually work throughout the year, or do you work seasonally, or only once in a while? | Throughout the year  Seasonally/part of the year  Once in a while |

**Observation of food intake**

1. Respondent’s relationship to child:
2. Did [NAME] eat and drink during the night after going to sleep and today before I arrived? Y/N

*If* “*yes*”*:*

- 1. What did he/she eat or drink? Add responses to the table

| **Variable** | **Response Options** |
| --- | --- |
| Meal type: | Breakfast, Lunch, Dinner, Snack, Other (indicate) |
| Location of food intake: | Home, Outside of home |
| Time of food intake: | Select time or add timestamp |
| Person administering food to child (relationship to child): | Select “Biological mother” or type in manually |
| Food/drink consumed: | Select food/ingredients from dropdown list or type in manually  Note: “Breastfeeding” will be an option in the dropdown |
| Person preparing the food: | Select “Biological mother” or type in manually |
| Notes (e.g., brand, breast milk from bottle or from breast directly) | Type in manually |
| Was the consumed amount more than “trivial” (i.e., more than one bite of a banana)? | Y/N/DK |

***NOTE:***

- ***THE CAPI WILL ALLOW TO ADD AS MANY OF THESE TABLES AS NEEDED.***
- ***THE OBSERVATION SHOULD END WHEN THE CHILD GOES TO SLEEP FOR THE NIGHT.***

**TIME ENDED: Timestamp**

**Multiple Pass Open Dietary Recall Questionnaire**

**DAY 2: Confirmation of eligibility**

**Observer name:**

**Date:**

**Day of week:**

**Location:**

**ID:**

**TIME BEGAN: Timestamp**

1. Did respondent provide verbal consent to participate? Y/N
2. Respondent’s relationship to child:
3. Did you feed the child yesterday? Y/N
4. Did anyone else feed the child yesterday? Y/N
   1. *If* “*yes*”: Are they available to participate in this interview? Y/N

| **TIME ENDED:** | **Timestamp** |
| --- | --- |
| ********************************************* |  |

**TIME BEGAN: Timestamp**

*Now I would like to ask you about liquids that [NAME] had yesterday during the day or the previous night (after going to sleep). Please tell me about all drinks, whether [NAME] had them at home or somewhere else.*

| **Question** | | **Response Option** |
| --- | --- | --- |
| 1. Was [NAME] breastfed yesterday during the day or the previous night (after going to bed)? | | Y/N/DK |
| *Yesterday during the day or the previous night, did [NAME] have…?* | |  |
| 1. Plain water? | | Y/N/DK |
| 1. Infant formula, such as [insert local names of common formula]?   ***[Insert skip pattern]*** | | Y/N/DK |
| If “*yes*”:   - 1. How many times did [NAME] drink formula? | | *If 7 or more, record “7”; if number of times unknown, record “9”* |
| 1. Milk from animals, such as fresh, tinned or powdered milk?   ***[Insert skip pattern]*** | | Y/N/DK |
| If “*yes*”:   - 1. How many times did [NAME] drink milk? | | *If 7 or more, record “7”; if number of times unknown, record “9”* |
| - 1. Was the milk or were any of the milk drinks a sweet or flavored type of milk? | | Y/N/DK |
| 1. Yogurt drinks such as [insert local names of common types of yogurt drinks]?   ***[Insert skip pattern]*** | | Y/N/DK |
| If “*yes*”:   - 1. How many times did [NAME] drink yogurt? | | *If 7 or more, record “7”; if number of times unknown, record “9”* |
| - 1. Was the yogurt or were any of the yogurt drinks a sweet or flavored type of yogurt drink? | | Y/N/DK |
| If “*yes*”:   - 1. How many times did [NAME] drink milk? | | *If 7 or more, record “7”; if number of times unknown, record “9”* |
| - 1. Was the milk or were any of the milk drinks a sweet or flavored type of milk? | | Y/N/DK |
| 1. Yogurt drinks such as [insert local names of common types of yogurt drinks]?   ***[Insert skip pattern]*** | | Y/N/DK |
| If “*yes*”:   - 1. How many times did [NAME] drink yogurt? | | *If 7 or more, record “7”; if number of times unknown, record “9”* |
| - 1. Was the yogurt or were any of the yogurt drinks a sweet or flavored type of yogurt drink? | | Y/N/DK |
| 1. Chocolate-flavored drinks including those made from syrups or powders? | | Y/N/DK |
| 1. Fruit juice or fruit-flavored drinks including those made from syrups or powders? | | Y/N/DK |
| 1. Sodas, malt drinks, sports drinks, or energy drinks? | | Y/N/DK |
| 1. Tea, coffee, or herbal drinks?   ***[Insert skip pattern]***  If “*yes*”:   - 1. Was the drink/ Were any of these drinks sweetened? | | Y/N/DK    Y/N/DK |
| 1. Clear broth or clear soup? | | Y/N/DK |
| 1. Any other liquids?   ***[Insert skip pattern]***  If “*yes*”:   - 1. What was the liquid or what were the liquids?   2. Was the drink or were any of these drinks sweetened? | | Y/N/DK    Y/N/DK |
| **TIME ENDED:** | **Timestamp** | |

***************************************************************

| **TIME BEGAN:** | **Timestamp** |
| --- | --- |
| *Now I would like to ask you about everything that [NAME] ate yesterday during the day or the previous night. I am interested in foods your child ate whether at home or somewhere else.* | |
| *Think about when [NAME] went to sleep the night before yesterday. Did (he/ she) eat anything during the night?*  **If “*yes*” ask:** *Please tell me everything [NAME] ate during the night.*  **Probe:** *Anything else?*  **Record answers using the food groups [listed in CAPI].** | |
| *Now think about when [NAME] woke up yesterday. Did (he/ she) eat anything at that time?*  **If “*yes*” ask:** *Please tell me everything [NAME] ate at that time.*  *What did [NAME] do after that? Did he/she eat anything at that time?*  **Repeat this series of questions, recording in the food groups, until the respondent tells you that the child went to sleep yesterday.**  **If a mixed dish is mentioned:**  **Probe:** *What were the main ingredients in [MIXED DISH]?*  **Record answers in the correct food groups [listed in CAPI].** | |
| **TIME ENDED:** | **Timestamp** |
| *************************************************************** |  |

|  |  |
| --- | --- |
| **TIME BEGAN:** | **Timestamp** |
| ***For each food group not mentioned after completing the above, ask:*** Just to make sure, did [NAME] eat [FOOD GROUP ITEMS] yesterday during the day or the night? |  |
| **Question** | **Response Option** |
| 1. Yogurt, other than yogurt drinks?   ***[Insert skip pattern]***  If “*yes*”:   - 1. How many times did [NAME] eat yogurt? | Y/N/DK |
| 1. Porridge, bread, rice, noodles, pasta, or [insert other commonly consumed grains from table A6.2, including foods made from grains like rice dishes, noodle dishes, etc.]? | Y/N/DK |
| 1. Pumpkin, carrots, sweet red peppers**,** squash, or sweet potatoes that are yellow or orange inside? [any additions to this list should meet “Criteria for defining foods and liquids as ‘sources’ of vitamin A” described in Box A6.1] | Y/N/DK |
| 1. Plantains, white potatoes, white yams, manioc, cassava, or [insert other commonly consumed starchy tubers or starchy tuberous roots that are white or pale inside from table A6.4]? | Y/N/DK |
| 1. Dark green leafy vegetables, such as [insert commonly consumed vitamin A-rich dark green leafy vegetables—see examples in table A6.5]? | Y/N/DK |
| 1. Any other vegetables, such as [insert commonly consumed vegetables from table A6.6]? | Y/N/DK |
| 1. Ripe mangoes, ripe papayas or [insert other commonly consumed vitamin A-rich fruits from table A6.7]? | Y/N/DK |
| 1. Any other fruits, such as [insert commonly consumed fruits from table A6.8]? | Y/N/DK |
| 1. Liver, kidney, heart or [insert other commonly consumed organ meats—see examples on table A6.9] | Y/N/DK |
| 1. Sausages, hot dogs, ham, bacon, salami, canned meat or [insert other commonly consumed processed meats—see examples on table A6.10]? | Y/N/DK |
| 1. Any other meat, such as beef, pork, lamb, goat, chicken, duck, or [insert other commonly consumed meat—see examples on table A6.11]**?** | Y/N/DK |
| 1. Eggs? | Y/N/DK |
| 1. Fresh fish, dried fish or shellfish? | Y/N/DK |
| 1. Beans, peas, lentils, nuts, seeds, or [insert commonly consumed foods made from beans, peas, lentils, nuts, or seeds]? | Y/N/DK |
| 1. Hard or soft cheese such as [insert commonly consumed types of cheese—see examples in table A6.16]? | Y/N/DK |
| 1. Sweet foods such as chocolates, candies, pastries, cakes, biscuits, or frozen treats like ice cream and popsicles, or [insert other commonly consumed sentinel sweet foods—see examples in table A6.17]? | Y/N/DK |
| 1. Chips, crisps, puffs, French fries, fried dough, instant noodles, or [insert other commonly consumed sentinel fried and salty foods—see examples in table A6.18]? | Y/N/DK |
| 1. Any other solid, semi-solid or soft foods?   If “*yes*”:   - 1. What was the food? [mark food group if it is not yet coded “yes”] | Y/N/DK |
| ***[Insert skip pattern]*** |  |
| 1. Did [NAME] eat any solid, semi-solid or soft food yesterday during the day or the previous night?   If “yes” probe:   1. What kind of solid, semi-solid or soft foods did [NAME] eat? [mark food group] | Skip 19 if at least a single “*yes*” is recorded in questions 1 through 18 the questionnaire ends; otherwise, it ends after question 19. |
| 1. How many times did [NAME] eat any solid, semi-solid or soft foods yesterday during the day or previous night? | If 7 or more, record “7”; if number of times unknown, record “9” |
| **TIME ENDED:** | **Timestamp** |

**Infant and Young Child Feeding Adaptations of the DQ-Q**

**DAY 2: Confirmation of eligibility**

**Observer name:**

**Date:**

**Day of week:**

**Location:**

**ID:**

|  |
| --- |

**TIME BEGAN: Timestamp**

1. Did respondent provide verbal consent to participate? Y/N
2. Respondent’s relationship to child:
3. Did you feed the child yesterday? Y/N
4. Did anyone else feed the child yesterday? Y/N
   1. *If* “*yes*”: Are they available to participate in this interview? Y/N

|  |  |  |
| --- | --- | --- |
| **TIME ENDED:** | **Timestamp** |  |
| *************************************************************** |  | |

**TIME BEGAN: Timestamp**

|  |
| --- |

**Module for Cambodia**

| **Sub Food Group** |  | | **Question** | **Response Option** | |  |
| --- | --- | --- | --- | --- | --- | --- |
| First 2 days after delivery | 1 | | In the first 2 days after delivery, was [NAME] given anything other than breastmilk to eat or drink—anything at all like water, infant formula, condensed milk, cinnamon water, or sugar water? | YES or NO | |  |
|  | Now I would like to ask you about liquids that [NAME] had yesterday during the day or at night. Please tell me about all drinks, whether [NAME] had them at home, or somewhere else.   Yesterday during the day or at night, did [NAME] drink: | | |  | |  |
| Water | 2 | | Plain water? | YES or NO | |  |
| Infant Formulas | 3 | | Infant formulas such as France Bebe, Dumex, Similax or Nutrilatt? | YES or NO | |  |
|  |  | | If “*yes*”:   1. How many times did (NAME) drink infant formula? (IF 7 OR MORE TIMES, RECORD '7'). | # | |  |
| Milk | 4 | | Milk from animals, including fresh, packaged or powdered? | YES or NO | |  |
|  |  | | If “*yes*”:   1. How many times did (NAME) drink milk? (IF 7 OR MORE TIMES, RECORD '7'). | # | |  |
|  |  | | If “*yes*”:   1. Was any of the milk a sweet or flavored type of milk? | YES or NO | |  |
| Soymilk and Nut Milks | 5 | | Soymilk, Lactasoy, or green bean milk? | YES or NO | |  |
|  |  | | If “*yes*”:   1. Was it a sweet or flavored type of drink? | YES or NO | |  |
| Chocolate- flavored/ sweetened drinks | 6 | | Chocolate frappe? | YES or NO | |  |
| Fruit juice | 7 | | Fruit juice, fruit drinks, sugarcane juice or fruit shake? | YES or NO | |  |
| Soft drinks | 8 | | Soft drinks such as Coca-Cola, Fanta, Sprite, Bacchus or M-150? | YES or NO | |  |
| Tea, coffee, herbal drinks | 9 | | Tea, coffee, or herbal drinks? | YES or NO | |  |
|  |  | | If “*yes*”:   1. Was the drink sweetened? |  | |  |
| Clear broth | 10 | | Clear broth or clear soup? | YES or NO | |  |
| Any other liquids | 11 | | Any other liquids? | YES or NO | |  |
|  |  | | If “*yes*”:   1. What was the drink? |  | |  |
|  |  | | 1. Was the drink sweetened? | YES or NO | |  |
|  | Now I would like to ask you about foods that [NAME] had yesterday during the day or at night. I am interested in foods your child ate whether at home or somewhere else. Please think about snacks and small meals as well as main meals.  I will ask you about different foods, and I would like to know whether your child ate the food even if it was combined with other foods. Please do not answer “yes” for any food or ingredient only used in a small amount to add flavor to a dish.   Yesterday during the day or at night, did [NAME] have: | | |  | |  |
| Yogurt/Yogurt drinks | 12 | | Yogurt? | YES or NO | |  |
|  |  | | If “*yes*”:   1. How many times did [NAME] have yogurt? | # | |  |
|  |  | | 1. Did [NAME] have any yogurt as a drink? | YES or NO | |  |
|  |  | | 1. Was it a sweet or flavored type of drink? | YES or NO | |  |
| Foods made from grains | 13 | | Rice, bobor krub kroeung or other porridge, Khmer rice pancake, Khmer noodles, glass noodles, bread, or corn? |  | |  |
| Vitamin A vegetables | 14 | | Carrots, pumpkin, or sweet potatoes that are orange inside? | YES or NO | |  |
| White roots/ tubers | 15 | | Potato, sweet potato, cassava, cassava noodles, taro, damlong daikla, or green banana? | YES or NO | |  |
| Dark Green Leafy Vegetables 1 | 16 | | Ivy gourd leaves, moringa leaves, green amaranth, water spinach, bok choy, or mustard greens? | YES or NO | |  |
| Dark Green Leafy Vegetables 2 | 17 | | Pumpkin leaves, sweet leaf bush, choy sum, spinach, kale, or broccoli? | YES or NO | |  |
| Other vegetables | 18 | | Eggplant, cauliflower, long beans, cabbage, bean sprouts, tomatoes, or okra? | YES or NO | |  |
|  | 19 | | Wax gourd, sponge gourd, bitter gourd, ridge gourd, bottle gourd, ivy gourd, or cucumber? | YES or NO | |  |
| Vitamin A-rich orange fruit | 20 | | Ripe mango, ripe papaya, or passion fruit? | YES or NO | |  |
| Other fruits | 21 | | Orange, mandarin, grapefruit, or pomelo? | YES or NO | |  |
|  | 22 | | Banana, watermelon, custard apple, pineapple, jackfruit, star fruit, or avocado? | YES or NO | |  |
|  | 23 | | Mangosteen, durian, rambutan, longan or langsat, guava, dragon fruit, or apple? | YES or NO | |  |
| Fish and shellfish | 24 | | Fish, seafood, eel, small shrimp, canned fish, or fermented fish? | YES or NO | |  |
| Organ Meats | 25 | | Kidney, heart, lung, liver or blood? | YES or NO | |  |
| Processed meat | 26 | | Sausages or ham? | YES or NO | |  |
| Meat | 27 | | Any other meat, such as beef, buffalo, pork, frog, wild animal, chicken, or duck? | YES or NO | |  |
| Eggs | 28 | | Duck eggs or chicken eggs? | YES or NO | |  |
| Legumes | 29 | | Soybeans, soymilk, peas, pigeon peas, red mung beans, or mung beans? | YES or NO | |  |
| Nuts and Seeds | 30 | | Peanuts, sunflower seeds, pumpkin seeds, or watermelon seeds? | YES or NO | |  |
| Cheese | 31 | | N/A | YES or NO | |  |
| Insects | 32 | | Crickets, bug bacon, cockroaches, snails, spiders, termites or grasshoppers? |  | |  |
| Sweets | 33 | | Cakes, donut, cookies, coconut sticky rice, sticky rice with fruit, sticky rice layer cake, or sweet sticky rice balls? | YES or NO | |  |
| Sweets | 34 | | Candy, chocolates, ice cream, lot svet, mung bean pudding, or coconut jellies? | YES or NO | |  |
| Savory and fried snacks | 35 | | Potato chips, shrimp chips, French fries, fried sweet potato, fried banana, or Instant noodles? |  | |  |
| Red palm oil | 36 | | N/A | YES or NO | |  |
|  |  |  | | |  | |
| [Adapted from the Global Diet Quality Project, (2021). Cambodia. [cited October 15, 2023.] Retrieved from: https://www.dietquality.org/countries/khm](file:///C:\Users\JenniferYourkavitch\Downloads\Adapted%20from%20the%20Global%20Diet%20Quality%20Project,%20(2021).%20Cambodia.%20%5bcited%20October%2015,%202023.%5d%20Retrieved%20from:%20https:\www.dietquality.org\countries\khm) in March, 2022.  Supported by USAID, the EU and BMZ (through GIZ), The Rockefeller Foundation, and SDC. | | | | | |  |

**Module for Zambia**

| **Sub Food Group** |  | **Question** | | **Response Option** |  |
| --- | --- | --- | --- | --- | --- |
| *First 2 days after delivery* | 1 | In the first 2 days after delivery, was [NAME] given anything other than breastmilk to eat or drink—anything at all like water, baby milk, or herbal concoctions? | YES or NO | |  |
|  | Now I would like to ask you about liquids that [NAME] had yesterday during the day or at night. Please tell me about all drinks, whether [NAME] had them at home, or somewhere else.   Yesterday during the day or at night, did [NAME] drink: | |  | |  |
| Water | 2 | Plain water? | YES or NO | |  |
| *Infant Formulas* | 3 | Baby milk such as Nan, Lactogen, S-26, or Nestum? | YES or NO | |  |
|  |  | If “*yes*”:   1. How many times did (NAME) drink infant formula? (IF 7 OR MORE TIMES, RECORD '7'). | # | |  |
| *Milk* | 4 | Cow or goat milk? | YES or NO | |  |
|  |  | If “*yes*”:   1. How many times did (NAME) drink milk? (IF 7 OR MORE TIMES, RECORD '7'). | # | |  |
|  |  | 1. Was any of the milk a sweet or flavored type of milk? | YES or NO | |  |
| *Soymilk and Nut Milks* | 5 | N/A | YES or NO | |  |
| *Chocolate- flavored/ sweetened drinks* | 6 | Hot chocolate or Milo, Milkit or Ama SipSip, or Cremora? | YES or NO | |  |
| *Fruit juice* | 7 | Juice? | YES or NO | |  |
| *Soft drinks* | 8 | Other drinks such as Coke, Sprite, Fanta, or energy drinks such as Monster, or Powerade? | YES or NO | |  |
| *Tea, coffee, herbal drinks* | 9 | Tea, coffee, or herbal drinks? | YES or NO | |  |
|  |  | If “*yes*”:   1. Was the drink sweetened? | YES or NO | |  |
| *Clear broth* | 10 | Clear broth or clear soup? | YES or NO | |  |
| *Any other liquids* | 11 | Any other liquids? | YES or NO | |  |
|  |  | If “*yes*”:   1. What was the drink? |  | |  |
|  |  | 1. Was the drink sweetened? | YES or NO | |  |
|  | Now I would like to ask you about foods that [NAME] had yesterday during the day or at night. I am interested in foods your child ate whether at home or somewhere else. Please think about snacks and small meals as well as main meals.  I will ask you about different foods, and I would like to know whether your child ate the food even if it was combined with other foods. Please do not answer “yes” for any food or ingredient only used in a small amount to add flavor to a dish.   Yesterday during the day or at night, did [NAME] have: | |  | |  |
| *Yogurt/Yogurt drinks* | 12 | Yogurt, sour milk, or mabisi? | YES or NO | |  |
|  |  | If “*yes*”:   1. How many times did [NAME] have yogurt, sour milk, or mabisi? | # | |  |
|  |  | 1. Did [NAME] have any yogurt, sour milk, or mabisi as a drink? | YES or NO | |  |
|  |  | 1. Was it a sweet [or flavored] type of drink? | YES or NO | |  |
| *Foods made from grains* | 13 | Nsima, sample, rice, bread, maize, macaroni, spaghetti, or porridge? | YES or NO | |  |
| *Vitamin A vegetables* | 14 | Carrots, pumpkin, butternut, or sweet potatoes that are orange inside? | YES or NO | |  |
| *White roots/ tubers* | 15 | Fresh cassava, roasted cassava, nshima from cassava, potato, or white sweet potato? | YES or NO | |  |
| *Dark Green Leafy Vegetables 1* | 16 | Ifisashi, pumpkin leaves, sweet potato leaves, bean leaves, cowpea leaves, or cassava leaves? | YES or NO | |  |
| *Dark Green Leafy Vegetables 2* | 17 | Spinach, Chinese, rape, amaranth leaves, lumanda, black jack, or other wild vegetable leaves? | YES or NO | |  |
| *Other vegetables* | 18 | Tomatoes, cabbage, impwa, eggplant, green pepper, mushrooms, or okra? | YES or NO | |  |
|  | 19 | Cucumber, green beans, beetroot, baby marrows, or cauliflower? | YES or NO | |  |
| *Vitamin A-rich orange fruit* | 20 | Ripe mango or pawpaw? | YES or NO | |  |
| *Other fruits* | 21 | Orange or tuma nut? | YES or NO | |  |
|  | 22 | Banana, apple, pear, guava, or avocado? | YES or NO | |  |
|  | 23 | Watermelon, pineapple, peaches, grapes, or strawberries? | YES or NO | |  |
| *Fish and shellfish* | 24 | Fish, kapenta, or chisense? | YES or NO | |  |
| *Organ Meats* | 25 | Offals, liver, kidney, or heart? | YES or NO | |  |
| *Processed meat* | 26 | Sausage, polony, bacon, biltong, or dried meat? | YES or NO | |  |
| *Meat* | 27 | Any other meat, such as cow meat, goat meat, sheep meat, pork, field mice, rabbit, or chicken? | YES or NO | |  |
| *Eggs* | 28 | Eggs? | YES or NO | |  |
| *Legumes* | 29 | Beans, peas, lentils, bambara nuts, or soya pieces? | YES or NO | |  |
| *Nuts and Seeds* | 30 | Groundnuts, peanut butter, peanut powder, sunflower seeds, pumpkin seeds, cashew nuts? | YES or NO | |  |
| *Cheese* | 31 | Cheese? | YES or NO | |  |
| *Insects* | 32 | Flying termites or caterpillars? | YES or NO | |  |
| *Sweets* | 33 | Any sweet foods such as cakes, biscuits, or donuts (ma donadi)? | YES or NO | |  |
| *Sweets* | 34 | Sweets, chocolates, ice cream or Freezit? | YES or NO | |  |
| *Savory and fried snacks* | 35 | Crisps, corn puffs, chips, fried cassava, fritters or vitumbuwa, fried fish, fried chicken, or instant noodles such as Eezee noodles? | YES or NO | |  |
| *Red palm oil* | 36 | N/A | YES or NO | |  |
|  |  |  |  | |  |
| [Adapted from Global Diet Quality Project. 2021. Zambia. [cited October 15, 2023] Retrieved from: https://www.dietquality.org/countries/zmb](file:///C:\Users\JenniferYourkavitch\Downloads\Adapted%20from%20%20Global%20Diet%20Quality%20Project.%202021.%20Zambia.%20%5bcited%20October%2015,%202023%5d%20Retrieved%20from:%20https:\www.dietquality.org\countries\zmb%20ww.dietquality.org.%202021) in May, 2022.  Supported by USAID, the EU and BMZ (through GIZ), The Rockefeller Foundation, and SDC. | | | | | |

| **TIME ENDED:** | **Timestamp** |
| --- | --- |

**Supplemental File 3. Costing Assessment**

For each recall method, we also calculated cost-efficiency and the average time to complete the questionnaire (based on CAPI timestamps) and to check data during field data collection. We calculated cost-efficiency as the total economic cost (USD) to collect, clean, and analyze data, divided by the number of participants per method. Costed activities included training, pilot-testing, data collection, data cleaning, and analysis.

To estimate personnel costs, we collected data on the time each study staff member spent per activity, differentiated by recall method (see table below). These data were based on method–specific personnel time use records, records of time spent on training and piloting per method, and recall. Personnel time was then valued using the wages or salaries of study staff. To estimate the opportunity cost of respondents’ time, we used the method-specific average time spent responding to the survey based on timestamps recorded on the CAPI forms. We also added an additional 10 minutes of time per respondent to each method to account for time spent providing informed consent. We approximated the value of respondents’ time using the national minimum wage in Cambodia and in Zambia as reported in the 2020–2021 International Labor Organization wage report in Table 4^(22)^.

We reported data on non-personnel expenditures, which included venues used for training, equipment, and supplies (laptops, tablets, photocopies, sim cards, etc.), transportation, respondent gifts, etc., based on study expense records. For expenditures on equipment with a useful life of longer than one year, we costed these items for use during the study assuming either local rental rates or based on their annualized cost including a 3% discount rate ^(23)^.

Average time to complete MDD questionnaire and field-based data checking

|  | Time to complete activity (mm:ss) | | | | | | |
| --- | --- | --- | --- | --- | --- | --- | --- |
|  | Cambodia (n=636) | | | | Zambia (n=608) | | |
| Activity | List-based | Multiple pass | Difference^‡^ | List-based | | Multiple pass | Difference |
| Average time per respondent to complete MDD-children questionnaire | 13:55 | 22:10 | 08:15 | 05:39 | | 16:54 | 11:15 |
| Average time per respondent for enumerators to check data before submitting to supervisor | 06:13 | 08:43 | 02:30 | Not applicable (n/a) ^§^ | | n/a | n/a |
| Average time per respondent for supervisors to check data in the field | 06:29 | 11:08 | 04:39 | n/a | | n/a | n/a |
| Total average time per respondent to complete the whole recall questionnaire and field-based data checking | 26:37 | 42:01 | 15:24 | n/a | | n/a | n/a |

‡^1^Difference calculated as time for multiple-pass method minus time for list-based method

§Time for field-based data checking was not captured in Zambia.
